# Exploring the relationship between cycle threshold values and oral manifestations in COVID-19: a comprehensive overview

**DOI:** 10.2340/aos.v83.41390

**Published:** 2024-09-23

**Authors:** Dalit Porat Ben Amy, Hanan Rohana, Maya Azrad, Michael V. Joachim, Ori Bar, Imad Abu El-Naaj, Avi Peretz

**Affiliations:** a Unit of Oral Medicine, the Baruch Padeh “Tzafon” Medical Center, Poriya, Israel; b Azrieli Faculty of Medicine, Bar-Ilan University, Safed, Israel; c Clinical Microbiology Laboratory, the Baruch Padeh “Tzafon” Medical Center, Poriya, Israel; d Unit of Oral and Maxillofacial Surgery, Shamir (Assaf ha-Rofeh) Medical Center, Tzrifin, Israel; e Department of Oral and Maxillofacial Surgery, the Maurice and Gabriela Goldschleger School of Dentistry, Faculty of Medicine, Tel Aviv University, Tel Aviv, Israel; f Department of Oral and Cranio-Maxillofacial Surgery, the Baruch Padeh “Tzafon” Medical Center, Poriya, Israel

**Keywords:** COVID-19, E gene, N gene, Ct values, Oral manifestations

## Abstract

**Objective:**

This cross-sectional study aimed to compare oral manifestations between severe acute respiratory syndrome coronavirus-2 (SARS-CoV-2)-positive and SARS-CoV-2-negative patients and to examine associations between oral symptoms, Ct values of E and N SARS-CoV-2 viral genes, and the implications of low Ct values indicating a high viral load, which is a predictive factor for the outcome of COVID-19.

**Methods:**

A total of 353 participating patients were aged ≥18 years with clinical manifestations of COVID-19 infection and tested for SARS-CoV-2 carriage at the medical center, by reverse transcriptase polymerase chain reaction (RT-PCR). All patients filled out an anonymous digital questionnaire regarding oral and general symptoms and overall medical health.

**Results:**

A significant association was found between SARS-CoV-2 carriage and dry mouth, unpleasant taste and changes in taste (*p* < 0.001); for example, 37.4% of the 147 SARS-CoV-2- positive participants had a dry mouth, compared to 18.9% of the 206 SARS-CoV-2- negative participants. Oral blisters were experienced by patients with an E gene Ct value of 10–20 (50%) or 21–30 (50%) (*p* = 0.041). Bad breath, dry mouth, unpleasant taste and changes in taste were mostly present in participants whose Ct values of both E and N genes were between 21 and 30.

**Conclusions:**

This study found significant associations between low Ct values of E and N SARS-CoV-2 viral genes and high viral load, indicating that Ct values can serve as predictive factors for COVID-19 outcomes. The findings suggest that while oral symptoms are present, the Ct values and associated high viral loads are more critical indicators of disease severity and prognosis.

## Introduction

An outbreak of severe acute respiratory syndrome (SARS) took place in Wuhan Hubei Province, China, in December 2019. Given its high phylogenetic similarity to the SARS coronavirus (SARS-CoV) identified in 2002, the virus was referred to as coronavirus-2 (SARS-CoV-2) and in February 2020, the associated infection was officially named coronavirus disease 2019 (COVID-19) by the World Health Organization [1].

The coronavirus is a positive-sense, single-stranded RNA virus named after the spiked, crown-like proteins expressed on its surface. These proteins are critical for viral activity, as they penetrate the invaded cells. Through changes to its protein structures, the virus hides or exposes areas that bind to the host cell [2].

A wide range of clinical manifestations have been reported, including gastrointestinal symptoms, weakness, dry cough, and dyspnea [3]. In addition, skin and oral mucosal manifestations have also been described, including erythematic rash, urticaria, and blisters [4], impaired taste (dysgeusia) and/or smell, ulcers, blisters, desquamate gingivitis, as well as oral pain and discomfort [5].

Reverse transcriptase polymerase chain reaction (RT-PCR) analysis of nasopharyngeal specimens is the gold standard method for detecting SARS-CoV-2 infection [6]. Several PCR assays have been developed, with the first targeting the E gene, which adheres to a membrane glycoprotein forming the viral envelope, and the N gene, which, along with the RNA genome, generates the nucleocapsid [7]. Determination of a positive test result is based on the PCR cycle threshold (Ct) value, which is the number of DNA amplification cycles needed to overcome a basal fluorescence threshold. Ct is associated with the target’s copy number in a sample, with low Ct values indicating a high viral load [8]. Aside from the Ct value of the viral genes determined in diagnostic labs, patient clinical data are valuable for understanding the patient’s infection status.

This study aimed to characterize the oral manifestations associated with COVID-19. To this end, the oral symptoms of participants with active COVID-19 were compared to those of SARS-CoV-2-negative patients, as reported via a digital questionnaire (D. Porat Ben Amy et al.). In addition, associations between the oral symptoms and the Ct values of viral genes, E and N were evaluated.

## Methods

### Study population

The study was designed as a questionnaire-based survey among participants aged ≥18 years who presented with clinical manifestations of COVID-19 infection and were tested for SARS-CoV-2 carriage at the medical center. Participants were divided into two groups based on their COVID-19 tests results (COVID-19 positive and COVID-19 negative). The study was approved by the medical center's Helsinki Committee (approval no. POR-0057-20). Each subject signed a consent form before enrolment. All methods were carried out in accordance with relevant guidelines and regulations.

### SARS-CoV-2 RNA detection by RT-PCR

Each participant provided a nasopharyngeal sample that was sent to the clinical microbiology laboratory for RT-PCR testing for presence of SARS-CoV-2. For this purpose, the nasopharyngeal sample (400 μl) was inactivated by addition of 350 μl enzymatic lysis buffer, followed by incubation for 30 min at 37°C. Viral RNA was first extracted from the inactivated sample using the QIAamp Viral RNA Kit (QIAGEN GmbH, Germany) and the QIAcube Automated Spin-column purification tool (QIAGEN GmbH), according to the manufacturer’s instructions. Before extraction, The Allplex 2019-nCOV Assay kit (Seegene, Seoul, South Korea) was used for SARS-CoV-2 RNA detection in patient samples and in negative and positive controls, according to the manufacturer’s instructions. The RT-PCR was performed in a BioRad CFX96 Real-Time Detection System (Hercules, CA, USA). A positive result was determined by the Ct values of the E and N genes, respectively [9], based on a scale of 0–40. Samples were tested in duplicates and the values presented are the average of the duplicates.

### Digital questionnaire

Following COVID-19 testing, all participants filled out an anonymous digital questionnaire prepared and sent by the Oral Medicine Unit at the medical center within 1 month of collection of the oropharyngeal sample, which requested subjective ratings of oral cavity characteristics, including the presence of oral ulcers and blisters, gingival sensitivity and bleeding, bad breath, unpleasant or changes in taste, dry mouth, and sore throat. Furthermore, the questionnaire included general questions concerning the patient’s medical condition, such as the presence of fever (body temperature >38°C), high blood pressure (HBP), heart diseases, smoking status, concomitant diabetes, and body mass index (BMI). The questionnaire was based on a validated questionnaire used in a previous study that evaluated oral health status indicators, initially developed for large-scale surveys of older adults. The study suggested that the indicators were very useful for descriptive oral health surveys in the general population [10].

### Statistical analysis

Variables are presented in absolute numbers and percentages. The Chi-squared test was used to compare the rates of oral symptom presence between SARS-CoV-2-positive and -negative participants. Linear regression was used to test the association between mean Ct value and number of symptoms. Multiple linear regression was used to test the association between mean Ct value and symptoms as potential predictors, using a stepwise selection method to minimize the number of predictors. The Chi-squared test for linear trend was used to analyze associations between specific Ct intervals and oral manifestations. All statistical analyses were conducted using R 4.0.1 (R Foundation for Statistical Computing, Vienna, Austria). Statistical significance was declared at *p*-values <0.05.

The sample size was calculated based on a Chi-squared test with a small-medium effect size (*W* = 0.2), power of 0.8, *p* = 0.05 and one degree of freedom. According to this calculation, 197 would be an appropriate sample size for the study.

## Results

### General clinical manifestations of SARS-CoV-2-positive compared to SARS-CoV-2-negative participants

This study included 353 participants who demonstrated clinical manifestations of COVID-19. A total of 147 (41.6%) participants tested positive for SARS-CoV-2 carriage, while the remaining patients were negative. The SARS-CoV-2-negative group included 118 (57.3%) males and 88 (42.7%) females, while the positive group included 74 (50.3%) males and 73 (49.7%) females. No associations were found neither between age subgroups (18–30, 31–49, 50–69 and >70 years) and test results (positive/negative for SARS-CoV-2). Furthermore, no associations were found between the test result and heart diseases, HBP, diabetes, smokers (>10 years), pregnancy or BMI. In contrast, a significant association was found between the test result and fever; among the SARS-CoV-2-negative participants, 38 (18.4%) had a fever, compared to 60 (40.8%) of the SARS-CoV-2-positive participants (*p* < 0.001).

### Oral symptoms of SARS-CoV-2-positive compared to SARS-CoV-2-negative participants

A significant association was found between the test result and dry mouth, unpleasant taste and changes in taste (*p* < 0.001 for each association) (Table 1). No associations were found for the other oral manifestations included in the questionnaire.

**Table 1:** A Summary of oral manifestations by SARS-CoV-2 carriage test results.

Characteristic	Negative (*N* = 206) *n* (%)	Positive (*N* = 147)*n* (%)	Total (*N* = 353) *n* (%)	*p*
**Oral ulcers**				
No	193 (93.7%)	134 (91.2%)	327 (92.6%)	0.369
Yes	13 (6.3%)	13 (8.8%)	26 (7.4%)	
**Oral blisters**				
No	203 (98.5%)	140 (95.2%)	343 (97.2%)	0.065
Yes	3 (1.5%)	7 (4.8%)	10 (2.8%)	
**Gingival sensitivity**				
No	181 (87.9%)	129 (87.8%)	310 (87.8%)	0.975
Yes	25 (12.1%)	18 (12.2%)	43 (12.2%)	
**Gingival bleeding**				
No	187 (90.8%)	133 (90.5%)	320 (90.7%)	0.924
Yes	19 (9.2%)	14 (9.5%)	33 (9.3%)	
**Bad breath**				
No	184 (89.3%)	124 (84.4%)	308 (87.3%)	0.168
Yes	22 (10.7%)	23 (15.6%)	45 (12.7%)	
**Dry mouth**				
No	167 (81.1%)	92 (62.6%)	259 (73.4%)	**<0.001**
Yes	39 (18.9%)	55 (37.4%)	94 (26.6%)	
**Unpleasant taste**				
No	186 (90.3%)	103 (70.1%)	289 (81.9%)	**<0.001**
Yes	20 (9.7%)	44 (29.9%)	64 (18.1%)	
**Changes in taste**				
No	184 (89.3%)	77 (52.4%)	261 (73.9%)	**<0.001**
Yes	22 (10.7%)	70 (47.6%)	92 (26.1%)	
**Sore throat**				
No	138 (67.0%)	93 (63.3%)	231 (65.4%)	0.468
Yes	68 (33.0%)	54 (36.7%)	122 (34.6%)	

### Associations between general clinical manifestations of COVID-19 and Ct values of E gene and N gene

To explore associations between the Ct and clinical COVID-19 manifestations, the Ct values of E gene and N gene were categorized into intervals of 10. No associations were found between the different Ct intervals of the N/E gene and heart diseases, HBP, diabetes, pregnancy or BMI. Furthermore, no association was found between the Ct value of the N/E gene and the place in which the participants were during the infection. For instance, concerning the N gene, 79.9% of the participants were at home during the infection, 14.3% at a motel, 3% at a hospital without ventilation, 1.5% at a hospital with ventilation, and 1.5% at another facility. A significant association was found between smoking (>10 years) and specific E gene Ct intervals (*p* = 0.026), with 45.45% of the smokers having a Ct value in the range of 10–20, 18.2% having a Ct value of 21–30 and 36.4% having a Ct of 31–40. Additionally, fever was associated with N gene Ct intervals (*p* = 0.021), with 25% of the SARS-CoV-2–positive participants reporting on fever showing a Ct of 10–20, 42.3% with a Ct of 21–30 and 32.7% with a Ct of 31–40.

### Associations between oral symptoms of COVID-19 patients and Ct values of E and N genes

Significant associations were found between specific Ct intervals of both genes and certain oral manifestations. For the E gene, oral blisters were seen in patients with a Ct value in the range of either 10–20 (50%) or 21–30 (50%) (*p* = 0.041) (Table 2). Gingival sensitivity (58.8%) and bleeding (53.8%) were mostly seen in patients with a Ct value of 21–30 (*p* = 0.006, *p* = 0.02, respectively). Sore throat was mainly associated with a Ct in the interval of 21–30 (*p* = 0.011). Additional manifestations correlating with Ct value were bad breath, dry mouth, unpleasant taste, and changes in taste (*p* < 0.001) (Table 2).

**Table 2:** A summary of oral manifestations of COVID-19 patients by Ct intervals of E gene.

Characteristic	E gene Ct intervals			
	10–20 (*N* = 23) *n* (%)	21–30 (*N* = 58) *n* (%)	31–40 (*N* = 52) *n* (%)	*p*
**Oral ulcers**				
No	20 (87.0%)	50 (86.2%)	51 (98.1%)	0.072
Yes	3 (13.0%)	8 (13.8%)	1 (1.9%)	
**Oral blisters**				
No	20 (87.0%)	55 (94.8%)	52 (100.0%)	**0.041**
Yes	3 (13.0%)	3 (5.2%)	0 (0.0%)	
**Gingival sensitivity**				
No	17 (73.9%)	48 (82.8%)	51 (98.1%)	**0.006**
Yes	6 (26.1%)	10 (17.2%)	1 (1.9%)	
**Gingival bleeding**				
No	18 (78.3%)	51 (87.9%)	51 (98.1%)	**0.021**
Yes	5 (21.7%)	7 (12.1%)	1 (1.9%)	
**Bad breath**				
No	12 (52.2%)	47 (81.0%)	51 (98.1%)	**<0.001**
Yes	11 (47.8%)	11 (19.0%)	1 (1.9%)	
**Dry mouth**				
No	5 (21.7%)	32 (55.2%)	48 (92.3%)	**<0.001**
Yes	18 (78.3%)	26 (44.8%)	4 (7.7%)	
**Unpleasant taste**				
No	6 (26.1%)	38 (65.5%)	49 (94.2%)	**<0.001**
Yes	17 (73.9%)	20 (34.5%)	3 (5.8%)	
**Changes in taste**				
No	3 (13.0%)	27 (46.6%)	41 (78.8%)	**<0.001**
Yes	20 (87.0%)	31 (53.4%)	11 (21.2%)	
**Sore throat**				
No	10 (43.5%)	33 (56.9%)	40 (76.9%)	**0.011**
Yes	13 (56.5%)	25 (43.1%)	12 (23.1%)	

For the N gene, oral ulcers (66.67%), gingival sensitivity (64.7%) and bleeding (53.85%) were mostly seen in patients with a Ct interval of 21–30 (*p* = 0.040, *p* = 0.004, *p* = 0.020, respectively) (Table 3). Sore throat was mainly associated with a Ct value of 21–30 (*p* = 0.014). Additional manifestations associated with N gene Ct value were bad breath, dry mouth, unpleasant taste, and changes in taste (*p* < 0.001).

**Table 3:** A summary of oral manifestations of COVID-19 patients by Ct intervals of N gene (D. Porat Ben Amy et al.).

Characteristic	N gene Ct intervals			
	10–20 (*N* = 20) *n* (%)	21–30 (*N* = 56) *n* (%)	31–40 (*N* = 57) *n* (%)	*p* value
**Oral ulcers**				
No	17 (85.0%)	48 (85.7%)	56 (98.2%)	**0.040**
Yes	3 (15.0%)	8 (14.3%)	1 (1.8%)	
**Oral blisters**				
No	18 (90.0%)	52 (92.9%)	57 (100.0%)	0.082
Yes	2 (10.0%)	4 (7.1%)	0 (0.0%)	
**Gingival sensitivity**				
No	15 (75.0%)	45 (80.4%)	56 (98.2%)	**0.004**
Yes	5 (25.0%)	11 (19.6%)	1 (1.8%)	
**Gingival bleeding**				
No	15 (75.0%)	50 (89.3%)	55 (96.5%)	**0.020**
Yes	5 (25.0%)	6 (10.7%)	2 (3.5%)	
**Bad breath**				
No	8 (40.0%)	46 (82.1%)	56 (98.2%)	**<0.001**
Yes	12 (60.0%)	10 (17.9%)	1 (1.8%)	
**Dry mouth**				
No	6 (30.0%)	26 (46.4%)	53 (93.0%)	**<0.001**
Yes	14 (70.0%)	30 (53.6%)	4 (7.0%)	
**Unpleasant taste**				
No	4 (20.0%)	35 (62.5%)	54 (94.7%)	**<0.001**
Yes	16 (80.0%)	21 (37.5%)	3 (5.3%)	
**Changes in taste**				
No	4 (20.0%)	23 (41.1%)	44 (77.2%)	**<0.001**
Yes	16 (80.0%)	33 (58.9%)	13 (22.8%)	
**Sore throat**				
No	7 (35.0%)	35 (62.5%)	41 (71.9%)	**0.014**
Yes	13 (65.0%)	21 (37.5%)	16 (28.1%)	

### Association between Ct value and number of oral symptoms

The ability to predict E gene and N gene Ct values by the number of oral symptoms was evaluated using a linear regression model. When no oral symptoms were present, the predicted mean Ct value was 36.43. Each additional oral symptom in the same patient lowered the Ct value by 2.37 units (*B* = −2.37, 95% CI [−2.78, −1.96], *p* < 0.001; Table 4). In addition, the number of oral symptoms explained 49.9% of the variance of the Ct value.

**Table 4:** Regression analysis of mean Ct value (of N and E genes) predicted by the number of oral symptoms (*N* = 133).

Predictors	B^1^	95% CI	*T*	*p*
**(Intercept)**	36.43	34.79 – 38.07	43.89	**<0.001**
**Number of symptoms**	−2.37	−2.78 to −1.96	−11.43	**<0.001**

1 B = unstandardized regression coefficients, CI = confidence intervals, *T* = *t*-value, *R*^2^ = 0.499

### Symptoms-based predictive model

**Figure 1:**
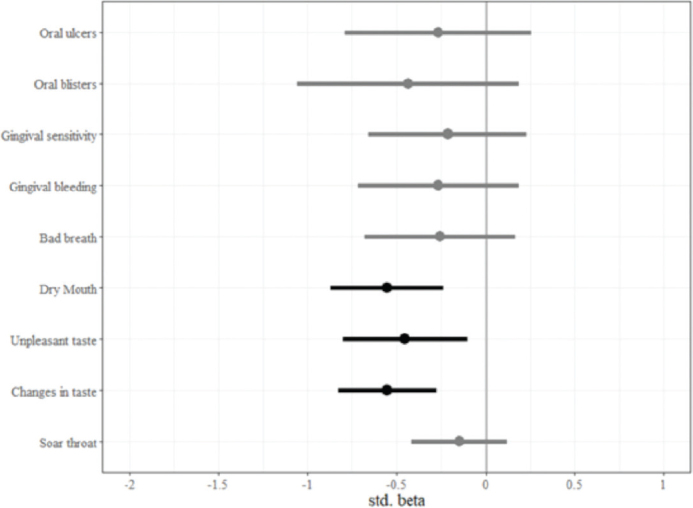
Final stepwise linear regression model for predicting mean N and E gene Ct values. This model was used to identify the symptoms that significantly predict Ct value. All nine symptoms in the questionnaire were included as independent variables and the mean Ct value as the dependent variable. Points represent standardized regression coefficients, bars represent 95% confidence intervals, black bars represent significant effects. Changes in taste had the strongest effect (β = −0.55), lowering the Ct value by 3.85 units, followed by dry mouth (β = −0.54) lowering the Ct value by 3.76 units.

A multiple linear regression model with stepwise selection was used to determine the minimal number of symptoms that significantly predicts Ct value of both genes. All nine symptoms rated in the questionnaire were included as independent variables and the mean Ct value served as the dependent variable. The final model included six symptoms explaining 53.3% of the variance in Ct value. Changes in taste had the strongest effect (β = −0.55), which lowered the Ct value by 3.85 units, followed by dry mouth (β = −0.54), which lowered the Ct value by 3.76 units (Figure 1).

## Discussion

This study categorized oral cavity symptoms in individuals with COVID-19 using an anonymous digital questionnaire that patients filled out after undergoing COVID-19 testing. The main objective was to investigate the connections between oral symptoms and the presence of SARS-CoV-2 as well as between oral symptoms and Ct values of the E and N genes.

At the beginning of the pandemic, fever was considered one of the main symptoms of SARS-CoV-2 infection. Yet, as the pandemic progressed, clinical caregivers found that some patients did not develop fever throughout the course of the infection [11]. Nevertheless, the current work found a significant association between fever and SARS-CoV-2 carriage, with 40.8% of the SARS-CoV-2-positive compared to 18.4% of the SARS-CoV-2-negative participants suffering from fever. Furthermore, a significant association was found between fever and Ct values of the N gene, with 67.3% of patients with fever showing a Ct interval <30. According to a previous work, the shorter the interval between sampling and symptom onset, the lower the Ct value [12], with lower Ct value indicating a higher viral load [13, 14]. Therefore, the present results suggest that most samples were taken close to the beginning of the infection and that higher viral load indeed yields fever symptom.

The three oral symptoms associated with SARS-CoV-2 carriage included dry mouth, unpleasant taste, and changes in taste. These findings align with earlier reports of dry mouth and taste disturbances as common symptoms of COVID-19 [15, 16], with dry mouth generally developing before other disease symptoms, suggesting it as an initial indicator of infection [17]. Furthermore, evaluating dry mouth does not necessitate medical procedures or interventions, making it a simple variable to incorporate into clinical questionnaires. Additionally, the link between taste disturbances and COVID-19 has been previously confirmed, indicating it can serve as a prognostic factor for the disease [18]. However, one cannot exclude the fact that olfactory impairment, which has also been associated with COVID-19 infection, may be the source of taste disturbances among some patients, challenging the precise determination of the chemosensation was clinically altered [14].

All the symptoms showing a significant association to Ct value were mostly observed in patients with E gene or N gene Ct of 10–20 or 21–30. These low Ct values indicate a high viral load, which has been consistently associated with severe disease and poorer outcomes [12]. This highlights the importance of Ct values as critical predictors of disease severity and prognosis in COVID-19 patients, overshadowing the role of oral symptoms as mere markers of infection onset [19].

The study investigated the link between oral COVID-19 symptoms and Ct values of the E and N genes, revealing that people with no oral symptoms had significantly higher Ct values (36.43), indicating lower viral loads. Each reported oral symptom corresponded to a decrease in Ct value by an average of 2.37 units, suggesting a higher viral load. This reinforces the critical role of Ct values and viral load as predictors of disease severity and outcomes in COVID-19 patients, highlighting their importance over the presence of oral symptoms. In simpler terms, more oral symptoms seemed to correlate with higher levels of the virus (as shown by lower Ct values). This suggests a potential connection between oral symptoms and increased viral load in COVID-19 patients.

The association between oral symptoms and lower Ct values observed in this study has important implications beyond simply identifying potential COVID-19 cases. Several studies have demonstrated that higher viral loads, as indicated by lower Ct values, are correlated with more severe disease progression and poorer clinical outcomes in COVID-19 patients [20, 21]. Our results suggest that the presence and number of oral symptoms may serve as an accessible indicator of potential disease severity, as these symptoms appear to correlate with higher viral loads. This could provide clinicians with an additional tool for risk stratification and early intervention in COVID-19 cases.

This study faced a few challenges: Tunnel vision: It only considered SARS-CoV-2 infection, even though other viruses could explain the symptoms. This makes it hard to definitively say SARS-CoV-2 caused them. Second-hand information: The study relied on people reporting their own symptoms, which might be inaccurate or influenced by how they interpreted their experience. Having healthcare professionals verify the information would have made it more reliable. Missing timeline: Although samples were collected after symptoms started, the exact stage of infection remained unknown. This is important because the virus might behave differently at various stages, affecting the results.

## Conclusion

In conclusion, this study provides insights into the relationship between oral symptoms and viral load in COVID-19 patients. While certain oral symptoms, particularly dry mouth and altered taste perception, appear to be associated with SARS-CoV-2 infection, their primary significance may lie in their correlation with lower Ct values, indicative of higher viral loads. Given the established link between viral load and disease severity, these oral manifestations could potentially serve as accessible indicators of disease progression risk. However, further research is needed to fully elucidate the prognostic value of oral symptoms in COVID-19. Our findings underscore the importance of considering oral health in the broader context of COVID-19 management and highlight the need for interdisciplinary approaches in understanding and treating this complex disease.

## Supplementary Material

Exploring the relationship between cycle threshold values and oral manifestations in COVID-19: a comprehensive overview
