# Supplementary material for: Exploring the relationship between cycle threshold values and oral manifestations in COVID-19: a comprehensive overview
Source: Acta Odontol Scand. 2024 Sep 23;83:41390. doi: 10.2340/aos.v83.41390 (PMC11443029; doi:10.2340/aos.v83.41390)
Supplement: Exploring the relationship between cycle threshold values and oral manifestations in COVID-19: a comprehensive overview [file AOS-83-41390-s1.pdf]

Supplementary material has been published as submitted. It has not been copyedited or typeset by Acta Odontologica Scandinavica.

COVID-19-positive questionnaire:

Questionnaire regarding oral symptoms during covid-19 disease:

Thank you for participating in the questionnaire.

This survey is part of a study conducted by the oral medicine unit.

This study aims to recognize the oral symptoms arising during the disease, in order to understand it better.

This is an anonymous questionnaire that may take 3 minutes of your time. You may answer part of the questions.

The leading researcher, got permission from the CEO of the medical center to conduct this study.

By answering this questionnaire, you agree to participate in the study.

- Please check below:

◎ Agree

◎ Disagree

- When were you tested positive for COVID-19?

(date)

- Where were you during the disease?

◎◎At home

◎ In a COVID-19 motel

◎ In the hospital without mechanical ventilation

◎ In the hospital with mechanical ventilation

◎ Somewhere else

- Gender:

◎◎Male

◎ Female

◎ I prefer not to say

- Were you pregnant at that time?

◎◎Yes

◎ No

- Which week of pregnancy?

(number)

- Your age:

◎◎18-30

◎ 31-49

◎ 50-69

◎ 70 and over

- Previous illnesses and risk factors:

◎◎Cardiovascular diseases

◎ Hypertension

◎ Diabetes Mellitus

◎ Smoking for 10 years or more

◎ Body Mass Index of 30 or more

◎ Hospitalization in the last 3 years (besides labor)

- Have you had a fever (over 37.5 degrees) before you tested positive and until recovery?

◎◎Yes

◎ No

- Did you have a sore throat at that time?

◎◎Yes

◎ No

The following questions are related to the oral symptoms in the 2 weeks before you were tested positive until recovery:

- Did you have mouth ulcers?

◎◎Yes

◎ No

- Did you have oral blisters?

◎◎Yes

◎ No

- Did you have gingival sensitivity?

◎◎Yes

◎ No

- Did you have bleeding gums?

◎◎Yes

◎ No

- Did you have bad breath?

◎◎Yes

◎ No

- Did you have dryness of mouth?

◎◎Yes

◎ No

- Did you have an unpleasant taste?

◎◎Yes

◎ No

- Did you have changes in your ability to taste?

◎◎Yes

◎ No

- any other strange oral symptoms during the COVID-19 disease?

Thank you for your time, Wishing you good health.

COVID-19-negative questionnaire:

We are approaching you since you had a COVID-19 test, that's result was negative.

Thank you for participating in the questionnaire.

This survey is part of a study conducted by the oral medicine unit in the Medical Center.

This study aims to recognize the oral symptoms arising during the disease, in order to understand it better.

This is an anonymous questionnaire that may take 3 minutes of your time. You may answer part of the questions.

The leading researcher got permission from the CEO of the medical center to conduct this study.

By answering this questionnaire, you agree to participate in the study.

- Please check below:

◎ Agree

◎ Disagree

- When were you tested for covid19?

(date)

- Gender:

◎◎Male

◎ Female

◎ I prefer not to say

- Were you pregnant at that time?

◎◎Yes

◎ No

- Which week of pregnancy?

(number)

- Your age:

◎◎18-30

◎ 31-49

◎ 50-69

◎ 70 and over

- Previous illnesses and risk factors:

◎◎Cardiovascular diseases

◎ Hypertension

◎ Diabetes Mellitus

◎ Smoking for 10 years or more

◎ Body Mass Index of 30 or more

◎ Hospitalization in the last 3 years (besides labor)

- Have you had a fever (over 37.5 degrees) prior to the test (not more than a week)?

◎◎Yes

◎ No

- Did you have a sore throat at that time?

◎◎Yes

◎ No

The following questions are related to the oral symptoms in the 2 weeks before you and 2 weeks after the test:

- Did you have mouth ulcers?

◎◎Yes

◎ No

- Did you have oral blisters?

◎◎Yes

◎ No

- Did you have gingival sensitivity?

◎◎Yes

◎ No

- Did you have bleeding gums?

◎◎Yes

◎ No

- Did you have bad breath?

◎◎Yes

◎ No

- Did you have dryness of mouth?

◎◎Yes

◎ No

- Did you have an unpleasant taste?

◎◎Yes

◎ No

- Did you have changes in your ability to taste?

◎◎Yes

◎ No

- any other strange oral symptoms during this period?

Thank you for your time, wishing you good health.
